# Pathologic Rate of Cancer After Surgery for Dysplasia Detected on Chromoendoscopy for Inflammatory Bowel Disease

**DOI:** 10.1093/crocol/otaf032

**Published:** 2025-06-05

**Authors:** Talal Dahab, Luca Stocchi, Amit Merchea, Dorin T Colibaseanu, Francis A Farraye, Kelly L Mathis, David A Etzioni, David H Bruining, Michael F Picco

**Affiliations:** Division of Colon and Rectal Surgery, Mayo Clinic, Jacksonville, FL 32224, USA; Division of Colon and Rectal Surgery, Mayo Clinic, Jacksonville, FL 32224, USA; Division of Colon and Rectal Surgery, Mayo Clinic, Jacksonville, FL 32224, USA; Division of Colon and Rectal Surgery, Mayo Clinic, Jacksonville, FL 32224, USA; Division of Gastroenterology and Hepatology, Mayo Clinic, Jacksonville, FL 32224, USA; Division of Colon and Rectal Surgery, Mayo Clinic, Rochester, MN 55905, USA; Division of Colon and Rectal Surgery, Mayo Clinic, Scottsdale, AZ 85054, USA; Division of Gastroenterology and Hepatology, Mayo Clinic, Rochester, MN 55905, USA; Division of Gastroenterology and Hepatology, Mayo Clinic, Jacksonville, FL 32224, USA

## Abstract

**Introduction:**

The objective of the study was to investigate the characteristics of patients who underwent surgery for dysplasia detected during chromoendoscopy (CE) surveillance for inflammatory bowel disease (IBD) and the incidence of cancer in the surgical specimen.

**Methods:**

A retrospective review of medical records of all patients with dysplasia on a background of underlying IBD diagnosed through CE was carried out at a tri-site enterprise tertiary referral center between 2006 and 2019. We aimed to assess the clinical characteristics of patients requiring surgery for dysplasia and the incidence of cancer in the surgical specimen.

**Results:**

Out of 219 patients with dysplasia on CE, 35 underwent surgery for dysplasia (16%). Indications for surgery were multifocal disease (*n* = 6), endoscopically unresectable lesions (*n* = 13), visible HGD (*n* = 7) and unifocal invisible LGD (*n* = 9). Out of 35 patients requiring surgery, 5 were found to have adenocarcinoma, one of whom with stage IIIB disease received postoperative chemotherapy. No patient with a pathologic diagnosis of adenocarcinoma had any evidence of recurrent disease after a mean postoperative follow-up of 32 months.

**Conclusions:**

While the incidence of cancer at the time of surgery for IBD-related dysplasia is not negligible, the rate of node-positive disease is low.

## Introduction

Patients with inflammatory bowel disease (IBD) of the large intestine are at increased risk of developing colorectal cancer. It is widely accepted that colorectal cancer surveillance programs are associated with a decreased incidence of cancer in the background of IBD when compared with patients without surveillance.^1^ Current guidelines from organizations such as the American College of Gastroenterology^2,3^ recommend the use of chromoendoscopy (CE) as one of the accepted adjuncts to white light colonoscopy (WLE) to optimize detection of dysplasia in patients with IBD.^4^

It is currently accepted that when dysplasia is endoscopically resectable, continued colonoscopic surveillance is preferable to surgery.^5^ However, in some cases, endoscopic management of dysplasia may not be possible and surgery becomes necessary. In some cases, the pathologic report following surgery with an endoscopic diagnosis of dysplasia reveals invasive adenocarcinoma. There is limited research on the risk of adenocarcinoma in patients who undergo surgery for dysplasia detected during CE surveillance.

The purpose of this analysis was to investigate the characteristics of patients who underwent surgery for dysplasia following CE surveillance for IBD and the incidence of cancer in the surgical specimen.

## Methods

This retrospective study was approved by the Institutional Review Board. It included adult patients who underwent CE at any site within the Mayo Clinic Enterprise between January 2006 and December 2019 and had a histologic diagnosis of colorectal dysplasia associated with IBD (including ulcerative colitis and Crohn’s disease) established during CE. Endoscopic procedures were performed by 22 experienced gastroenterologists with an interest in IBD distributed through the 3 main Mayo Clinic Enterprise sites. The diagnosis of dysplasia associated with IBD was established by experienced pathologists and confirmed by a second pathologist, as necessary.

A retrospective chart review of medical records collected demographic information, specific IBD diagnosis, disease duration from the time of diagnosis, smoking history, coexisting primary sclerosing cholangitis, family history of colorectal cancer (CRC), and endoscopic and histologic findings on CE. The aspect of the lesions (polypoid vs non-polypoid) and the adequacy of endoscopic excision (complete or incomplete) were based on the endoscopist’s report. Targeted biopsies were collected during CE at the endoscopist’s discretion and were obtained in all cases except for one patient who was poorly tolerant of colonoscopy. Histological grading of lesions was categorized as negative for dysplasia, indefinite for dysplasia, low-grade dysplasia (LGD), and high-grade dysplasia (HGD). The location of dysplastic lesions was recorded by dividing the colon into four segments: cecum/ascending colon, transverse colon, descending colon, and rectum/sigmoid colon. Details on the presence and location of possible active inflammation of the colorectal mucosa at the time of chromoendoscopy were not retrospectively available. We compared patient and disease characteristics between patients who underwent CE without surgery and those who required surgery for dysplasia. Surgical outcomes, including the indication for surgery, initial operation performed, surgical pathology findings, and presence of invasive carcinoma in the surgical specimen, were also recorded. Patients who had a preoperative diagnosis of colorectal cancer, those who underwent surgery for indications other than IBD-associated dysplasia, patients who did not undergo CE, or those without endoscopic biopsy consistent with dysplasia on the background of IBD colitis were excluded from the study. In cases where surgery was not deemed necessary, patients were followed up with regular surveillance endoscopies or CE based on the treating gastroenterologist’s preference. Our clinicians recommended surgery according to the accepted guidelines except for individuals who were considered poor surgical candidates.

CE is performed at our institution by spraying the colon with a 0.2% FD&C Blue #2 solution (chemically similar to indigo carmine, Professional Compounding Centers of America, Houston, TX, USA). The same endoscopist did not always perform the follow-up CE.

Any discrepancies in the information collected were resolved through review by multiple researchers and/or consulting the medical record. Statistical analysis was performed on SPSS software (version 25.0; IBM Corp, Armonk, NY). Standard descriptive statistics were obtained and presented as percentages, mean ± SD.

## Results

**Figure 1.**
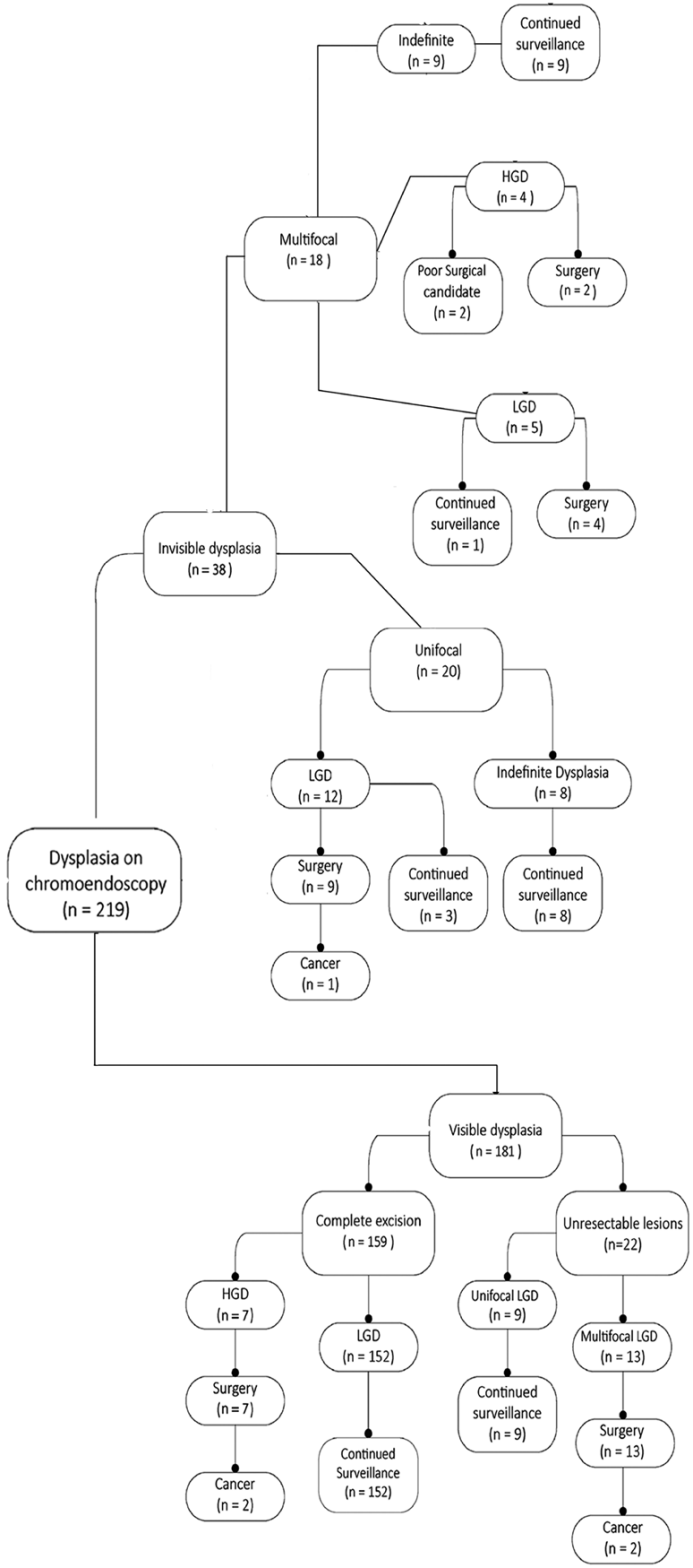
Number of patients with dysplasia, types of dysplasia, and number of patients undergoing surgery

A total of 392 patients underwent CE, of whom 219 patients were found to have dysplasia on endoscopic biopsy. Figure 1 demonstrates the number of patients with dysplasia, types of dysplasia and those who underwent surgery. Two patients with HGD were considered poor surgical candidates due to their comorbidities and were therefore recommended against surgery. Ten patients refused surgery, recommended for unresectable unifocal LGD and multifocal invisible LGD in 9 and one case, respectively.

Ultimately, 35 patients underwent surgery for dysplasia. The indications for surgery were multifocal disease in 6 patients, endoscopically unresectable lesions in 13 patients, unifocal invisible LGD in 9 patients, and HGD in 7 patients.

Demographics and other characteristics of the 35 patients who underwent surgery are reported in Table 1. Most of the patients had a baseline history of UC. The meantime from IBD diagnosis to the first CE was 21.4 years, while the meantime from IBD diagnosis to surgery was 22.6 years. Since their diagnosis and before surgery, patients had undergone a mean of 6.0 colonoscopies (range: 1-6), and 2.1 CEs (range: 1-6). A total of 29 patients had a history of previous endoscopic excision of visible and histologically confirmed dysplasia lesions during their endoscopic surveillance preceding surgery. The mean number of endoscopic excisions carried out prior to referral to our institution was 3 (range: 0-7), out of which a mean of 2 (range: 0-4) had been performed elsewhere. Patients had an average of 2 sites of dysplasia per session (range, 0-7). Full details of endoscopic findings can be found in Table 2.

**Table 1.** Demographics, patients, and disease characteristics (*n* = 35)

Females	19
Age	57.8 (16.2)
Smoking status	
Current	0
Former	9
Never	26
Primary sclerosing cholangitis	16
Family history of colorectal cancer	3
Inflammatory bowel disease diagnosis	
Ulcerative colitis	24
Crohn’s disease	10
Indeterminate colitis	1
IBD duration at the time of first chromoendoscopy (years)	21.4 (15.1)
Known dysplasia before chromoendoscopy	34
Number of endoscopic procedures before surgery^a^	
Colonoscopy	6 (1-6)
Chromoendoscopy	2.1 (1-6)
Endoscopic excisions	3 (0-7)

^a^Number (range), otherwise data reported as mean (± SD).

**Table 2.** Endoscopic and histopathology findings (85 lesions in 35 patients undergoing surgery)

Dysplasia morphology (*n* = 85)	
Invisible	22
Visible	63
Polypoid	58
Non-polypoid	5
Endoscopic location of visible dysplasia (*n* = 63)	
Cecum/ascending colon	18
Transverse colon	9
Descending colon	6
Sigmoid colon	20
Rectum	10
Dysplasia multifocality (*n* = 35)	
Unifocal	16
Multifocal	19
Grade of dysplasia (*n* = 35 patients)	
Low-grade	26
High-grade	9
Location of dysplastic sites (*n* = 85)	
Cecum/ascending colon	23
Transverse colon	12
Descending colon	10
Sigmoid colon	26
Rectum	14
Sporadic adenoma in grossly preserved mucosa	6

Data reported as mean (± SD) or number (%).

The indications for surgery, operations performed, and surgical findings are reported in Table 3. The most common type of procedure performed was total proctocolectomy, associated with the creation of end ileostomy or, less frequently, ileal pouch-anal anastomosis. The specimen’s pathologic examination revealed no dysplasia or cancer in 7 patients, dysplasia in 23 patients (LGD in 16 and HGD in 7 patients, respectively), and invasive cancer in 5 patients. One patient’s surgical specimen contained invasive carcinoma associated with a synchronous area of LGD. Cancer rates based on the preoperative dysplasia grades were 11.6% (3/26) for LGD and 22.2% (2/9) for HGD. Only one patient had stage 3 disease and completed postoperative chemotherapy with FOLFOX regimen (folinic acid, fluorouracil, and oxaliplatin). No patient had evidence of cancer recurrence after a mean postoperative follow-up of 32 months. Two patients underwent delayed completion proctectomy after initial total abdominal colectomy, and their proctectomy specimens did not indicate any additional dysplasia. Patients undergoing surgery were associated with a significantly longer duration of their disease and a higher number of performed colonoscopies. Other patient and disease characteristics were not statistically different (Table 4).

**Table 3.** Surgical variables and specimen pathology findings

Operation performed (*n* = 35)	
Total proctocolectomy and end ileostomy	13
Total proctocolectomy and J-pouch anal anastomosis	10
Total abdominal colectomy and ileorectal anastomosis	5
Total abdominal colectomy and end ileostomy	3
Proctectomy and colostomy/colectomy and ileostomy	2
Segmental colectomy and primary anastomosis	2
Patients with no dysplasia or cancer	7
Patients with dysplasia on surgical pathology	23
Dysplasia on surgical pathology (*n* = 23)	
Unifocal	13
Multifocal	10
Highest grade of dysplasia (*n* = 23)	
Low-grade	16
High-grade	7
Indefinite	0
Total number of sites with dysplasia on specimen pathology	35
Location of dysplasia (*n* = 35)	
Cecum/Ascending colon	9
Transverse colon	6
Descending colon	5
Sigmoid colon	5
Rectum	10
Adenocarcinoma staging (*n* = 5)	
Stage 1	1 (T1 N0)
Stage 2	3 (T3 pN0)
Stage 3	1 (T3 N1b)
Adenocarcinoma location (*n* = 6 in 5 patients)	
Cecum/Ascending colon	3
Transverse colon	1
Descending colon	0
Sigmoid colon	1
Rectum	1

1 patient had 2 synchronous adenocarcinomas, 7 patients had multifocal LGD, 4 patients had multifocal HGD, no patients had combination of HGD and LGD.

**Table 4.** Comparison of patient and disease characteristics between patients who underwent surgery and patients who did not undergo surgery

	No surgery (*N* = 357)	Surgery (*N* = 35)	Total (*N* = 392)	*P* value
Patient age	62.5 (21.6, 90.7)	61.2 (31.9, 79.6)	62.4 (21.6, 90.7)	0.28
Female Gender	143 (40.1%)	14 (40.0%)	157 (40.1%)	1.00
Smoking status*				
Current	18 (5.1%)	0 (0.0%)	18 (4.6%)	0.28
Former	116 (32.6%)	9 (25.7%)	125 (32.0%)	
Never	222 (62.4%)	26 (74.3%)	248 (63.4%)	
History of PSC	75 (21.0%)	12 (34.3%)	87 (22.2%)	0.087
Family history of colorectal cancer	54 (15.2%)	3 (8.6%)	57 (14.6%)	0.45
IBD duration (mo) upon first CE	161.9 (0.1, 826.5)	198.0 (19.2, 640.1)	166.3 (0.1, 826.5)	0.041
Known dysplasia before CE	325 (91.0%)	31 (88.6%)	356 (90.8%)	0.55
Number of colonoscopies	6.0 (1.0, 45.0)	5.0 (1.0, 20.0)	6.0 (1.0, 45.0)	0.026
Number of CE procedures	2.0 (1.0, 8.0)	1.0 (1.0, 6.0)	2.0 (1.0, 8.0)	0.11
Number of endoscopic resections	1.0 (0.0, 46.0)	1.0 (0.0, 15.0)	1.0 (0, 46.0)	0.90

Data presented as median (range) or number (%). IBD: Inflammatory bowel disease; CE: chromoendoscopy; PSC: Primary sclerosing cholangitis. * missing data in one patient.

## Discussion

The present study found that the risk of advanced colorectal cancer with lymph node involvement among patients who underwent surgery for dysplasia associated with IBD after CE was low and there was no evidence of cancer recurrence after over 2 and half years of postoperative follow-up. This study provides contemporary data focusing on one of the accepted standards of care for IBD surveillance and contemporary surgery. Its results corroborate the continued use of endoscopic management for IBD-associated dysplasia when feasible. All patients in this study had at least one CE procedure and access to qualified endoscopists experienced in advanced interventional procedures. It is therefore not surprising that patients undergoing surgery were associated with significantly longer disease duration and number of previous colonoscopies, often times entailing endoscopic excision of dysplasia before their ultimate referral for surgery, reflecting the modern multidisciplinary approach to the management of IBD-associated dysplasia.^6^ In addition, our dysplasia detection rate of 56% is substantially higher than the 20%-30% previously reported.^7^ When considering CE, a review based on randomized trials reported a dysplasia detection rate of 16%^8^ and a single institutional experience outside of randomized trials reported a dysplasia detection rate of 28% and the corresponding proportion of patients requiring colectomy of 2.6%.^9^ Both the high dysplasia detection rate and a colectomy rate of 9% associated with our series might depend on patient selection bias associated with a specialized tertiary referral center, where over 90% of patients undergoing CE had a preexisting diagnosis of IBD-associated dysplasia. On the other hand, our study population also included 7 patients undergoing surgery for dysplasia who however had no evidence of dysplasia or cancer in their surgical specimen. This is a well-known phenomenon as a systematic review examining 12 surgical cohort studies of 450 patients indicated a rate of absent dysplasia in the surgical specimen of 37%.^10^ Most of the included studies did not use advanced videoendoscopy techniques, which might explain the discrepancy with our own data. In this respect, it has also been suggested that with modern interventional techniques a substantial proportion of index lesions might end up being completely removed endoscopically before surgery.^11^

Earlier studies have reported high rates of invasive adenocarcinoma in patients undergoing surgery for dysplasia associated with IBD, with rates as high as 19% for LGD and 42% for HGD.^12–14^ The lower rate of cancer observed in the current study reflects the effectiveness of endoscopic management of IBD-associated dysplasia, as most patients in the study were able to undergo multiple endoscopic procedures to remove dysplasia before being referred for surgery. It is worth noting that the risk of invasive cancer based on the degree of dysplasia in endoscopic biopsies is just one of the factors to consider, as it is no longer believed that an area of LGD necessarily indicates an increased risk of cancer throughout the entire colorectal mucosa. Previous research from our institution showed that 3% of patients with HGD identified through surveillance biopsies had undetected cancer.^15^ In a pooled analysis of 33 studies on IBD colitis, the rate of incidental synchronous cancer found at colectomy for preoperative diagnoses of visible HGD, invisible HGD, visible LGD, and invisible LGD were 13.7%, 11.4%, 2.7%, and 2.4%, respectively.^16^ Other factors that may influence the risk of cancer but have not been extensively studied include multifocal dysplasia, which was the most common indication for surgery in our study, the inability to endoscopically resect the area of dysplasia, and the presence of a synchronous stricture. It is notable that ten patients did not undergo surgery despite having clear indications for surgery according to current guidelines, such as multifocal invisible dysplasia and incomplete endoscopic resection.^17^ It was difficult to retrospectively clarify the details included in the electronic medical chart of the discussions between patients and clinicians on management of dysplasia. Given the uniform support of current guidelines among our clinicians, we therefore assumed that all patients who did not have surgery according to guidelines did refuse surgery. This was an assumption we were comfortable with, given the recognition that some patients are willing to accept a higher risk of cancer in order to avoid surgery.^18^ In one survey specifically focused on IBD-associated dysplasia, 60% of patients stated they would refuse a physician recommendation for elective colectomy despite being told that they have a 20% risk of cancer now.^19^ Further studies are needed to better understand the consequences and actual incidence of cancer after refusal of surgery in these cases. Our study was restricted to contemporary patients who underwent CE, allowing us to select a homogeneous patient population that received the best available endoscopic treatments before undergoing surgery. CE can overcome the well-known limitations of traditional, pre-videoendoscopic WLE surveillance,^20^ and several studies have shown its advantages.^21,22^ However, more recent evidence suggests that high-definition WLE and virtual CE with targeted biopsies are acceptable equivalents to CE,^11,23^ so that different contemporary techniques are considered as comparable in what is referred to as “the videoendoscopic era.”^16^ The current study has limitations, as it was designed to assess patients who required surgery for the management of IBD-associated dysplasia despite the use of CE and access to advanced endoscopic techniques. It was therefore unable to assess all aspects of CE as a surveillance strategy, and we did not longitudinally assess patients undergoing endoscopic surveillance after surgery. As a result, it is also impossible based on the present study to assess the potential failure of endoscopic surveillance in cases where a patient develops biopsy-proven invasive adenocarcinoma during multiple serial CE procedures. In addition, the study was conducted at a tri-site referral center, but the sample size was relatively small. We could have included additional patients requiring surgery for IBD-associated dysplasia detected using other endoscopic approaches, but this would have further increased the heterogeneity of our patient population. The study was retrospective in nature and relied on electronic medical records, which may be subject to incomplete or inaccurate data. Moreover, it is important to note that the present study was conducted at a tertiary referral center with a focus on the management of IBD, and it is possible that some patients may have been referred with more advanced lesions diagnosed elsewhere. A previous meta-analysis of 18 studies including 1037 patients found a pooled risk of colorectal cancer of 2 per 1000 years of patient follow-up after endoscopic resection of dysplasia,^24^ while in our own study population the incidence of cancer in the specimen was not low. Despite this potential referral bias, the mean number of CE procedures undergone by the patients in our study was 2, in addition to multiple other WLE procedures, and we believe that our patient population underwent surgery only after all endoscopic options had been exhausted.

Another limitation of our study is the lack of information on whether the dysplasia associated with adenocarcinoma was detected during an index or surveillance endoscopy. However, it is worth noting that the majority of patients in our study had a history of dysplasia detected on previous white light endoscopy, indicating that the dysplasia was likely detected during surveillance rather than an index endoscopy.

In conclusion colorectal cancer in the surgical specimen of patients requiring surgery for IBD-associated dysplasia detected on CE is a recognized risk, but a locally advanced cancer with lymph node involvement was rare.

## Data Availability

The data underlying this article will be shared on a reasonable request to the corresponding author.
